# Detection of latent brain states from spontaneous neural activity in the amygdala

**DOI:** 10.1371/journal.pcbi.1012247

**Published:** 2025-02-13

**Authors:** Alexa Aucoin, Kevin K. Lin, Katalin M. Gothard

**Affiliations:** 1 Program in Applied Mathematics, University of Arizona, Tucson, Arizona, United States of America; 2 Department of Mathematics, University of Arizona, Tucson, Arizona, United States of America; 3 Department of Physiology, University of Arizona, Tucson, Arizona, United States of America; University of Edinburgh, UNITED KINGDOM OF GREAT BRITAIN AND NORTHERN IRELAND

## Abstract

The amygdala responds to a large variety of socially and emotionally salient environmental and interoceptive stimuli. The context in which these stimuli occur determines their social and emotional significance. In canonical neurophysiological studies, the fast-paced succession of stimuli and events induce phasic changes in neural activity. During inter-trial intervals, neural activity is expected to return to a stable and featureless level of spontaneous activity, often called baseline. In previous studies we found that context, such as the presence of a social partner, induces brain states that can transcend the fast-paced succession of stimuli and can be recovered from the spontaneous, inter-trial firing rate of neurons. Indeed, the spontaneous firing rates of neurons in the amygdala are different during blocks of gentle grooming touches delivered by a trusted social partner, and during blocks of non-social airflow stimuli delivered by a computer-controlled air valve. Here, we examine local field potentials (LFPs) recorded during periods of spontaneous activity to determine whether information about context can be extracted from these signals. We found that information about social vs. non-social context is present in the local field potential during periods of spontaneous activity between the application of grooming and airflow stimuli, as machine learning techniques can reliably decode context from spectrograms of spontaneous LFPs. No significant differences were detected between the nuclei of the amygdala that receive direct or indirect inputs from areas of the prefrontal cortex known to coordinate flexible, context-dependent behaviors. The lack of nuclear specificity suggests that context-related synaptic inputs arise from a shared source, possibly interoceptive inputs, that signal the physiological state of the body during social and non-social blocks of tactile stimulation.

## Author summary

The amygdala responds to a large array of socially and emotionally salient stimuli, both external (e.g., affective or neutral touch) and internal (e.g., heart rate), and the context in which these stimuli are processed. It thus plays an important role in how social animals like primates interact. Recent work showed that individual cells in the amygdala carry contextual information during the periods between stimuli, characterized by spontaneous baseline activity, i.e., socially significant interactions lead to detectable, persistent changes in activity in the amygdala. Here, we show that using modern machine learning techniques, we can identify, during this period of spontaneous activity, context-related signals not only from the individual neurons but also from the collective activity of large groups of brain cells. As a central task of computational neuroscience is to identify cognitive states and their neural and behavioral correlates, this study hints at how contextual information is encoded in the amygdala and contributes to the growing understanding of the potentials and pitfalls of machine learning methods in neuroscience.

## Introduction

During natural engagement with the environment, the brain concomitantly processes specific stimuli pertinent to an event and the context in which the event occurs. Stimulus parameters, internal transformations, and the resulting behaviors can be decoded from the activity of ensembles of simultaneously active neurons, and local field potentials (LFPs). Decoding context, however, is challenging because in most experimental settings, context is unchanging and often conflated with stimulus-evoked neural activity. Context signaling is more likely confined to spontaneous activity during inter-trial periods, often referred to as baseline activity. The baseline or spontaneous activity of individual neurons and neural populations varies with the context. For example, when rats learned the probability of a predator interfering with their run toward a coveted reward, the spontaneous activity in the amygdala exhibits correlations with the likelihood of an encounter with the predator. While spontaneous firing rates vary in proportion to animals’ anticipatory anxiety, the predator-induced firing rates remain unchanged [1]. In a similar vein, when monkeys learn to associate odors with positive and negative outcomes, the spontaneous firing rate of neurons in the amygdala and anterior cingulate cortex retain information about the strength of the learned association during the time intervals between trials [2]. Beyond the amygdala and affective states, the spontaneous activity of neurons in the neocortex and the basal ganglia can retain information about the outcome of numerous preceding trials that contribute to predictions for upcoming trials [3]. Likewise, spontaneous neural activity in the marmoset prefrontal cortex preceding and following a perceived vocalization predict the likelihood of a reciprocating response [4].

It appears, therefore, that neural activity during periods of spontaneous activity might be fertile ground to explore how the brain integrates context and stimuli across multiple time scales, how it predicts – rather than reacts to – external events, and how it creates persistent affective states. Indeed, affective states, such as anxiety, persist longer than an emotional reaction to the stimuli that predict negative outcomes. (Affect refers to coordinated brain-body states described by valence − pleasant or unpleasant − and arousal, i.e., a degree of engagement with the outside world [5]. Emotions are more complex mental states anchored to affect and often experienced as feelings.) We have recently demonstrated that grooming, the most common form of social and affective touch in macaques, elicits persistent changes in the spontaneous firing rate in 25–45% of neurons in the amygdala [6]. The observed changes in spontaneous activity were correlated with the animal’s physiological state (low sympathetic or high parasympathetic tone) and with the social context. The presence of the groomer near the monkey, even in the absence of grooming gestures, was sufficient to shift the spontaneous activity in the direction in which grooming would shift it. However, this study was focused on the spontaneous firing rates of individual neurons and left open the possibility that the LFPs, that represent the joint activity of neural populations at the mesoscale level, contain comparable information about the brain state of the animal. This is significant because the presence of such information in the LFPs would indicate that contextual information is not just carried by select neurons but reflects a wider state change in the relevant circuitry. From an experimental point of view, LFPs are more robust than single units and less prone to processing artifacts. In this paper, we examine the encoding of contextual information in LFPs recorded during the inter-trial period, focusing on two main questions.

First, we asked whether social context can be decoded from LFPs recorded from the amygdala during inter-trial intervals (spontaneous activity). The large fraction of neurons (25–45%) that showed context-dependent changes in spontaneous firing rates give rise to a specific covariance pattern across a population of neurons. That is, subsets of these neurons can be grouped together based on how they modulate their firing rates (e.g. suppressed, elevated, bursts, etc.) and the coactivity groupings of these subsets persists across repeated experimental epochs. Such covariance patterns have been detected in both single unit activity and LFPs [7] but only during engagement with a stimulus or a task parameter. We hypothesize that these coactivity patterns, which we sometimes refer to as “latent network dynamics,” are context-dependent, can persist across trials, and can be decoded from spontaneous activity.

Second, we asked whether there is a detectable difference in data recorded from different nuclei of the amygdala. We hypothesized that context-related activity will be most prominent in the basal and accessory basal nuclei that receive more robust inputs from the prefrontal cortex than the lateral and central nuclei [8–11]. As the LFP in each nucleus (subjected to common reference averaging to eliminate volume-conducted components), results from the synaptic currents received and summed across thousands of neurons [12,13], the LFP in the basal and accessory basal nuclei may be driven by inputs from the primate-specific areas of the prefrontal cortex that enable context-dependent, flexible emotional behaviors [14]. Alternatively, the context-related activity may arise from interoceptive inputs, signaling to the brain the parasympathetic-dominated physiological state observed during the grooming blocks [6]. Based on traditional anatomical studies in non-human primates, the interoceptive signals are transmitted to the amygdala both directly from the brainstem and via the insula [15,16]. The inputs from the brainstem and hypothalamus target primarily the central nucleus (as the central nucleus establishes reciprocal processing loops with autonomic effectors). The inputs from the insula target both the central nucleus and the nuclei of the basolateral complex. If the interoceptive signals are the primary driver of spontaneous activity, and these signals are more broadly distributed across the nuclei of the amygdala than prefrontal signals, we expect comparable spontaneous LFP patterns during the inter-stimulus periods in all nuclei of the amygdala. Note that we used the term “spontaneous” instead of “baseline” activity to distance ourselves from the idea that between the presentations of stimuli the brain returns to the same ‘contentless’ state, and that the content of brain states is related primarily to the organism’s engagement with external stimuli. Although the term “spontaneous” suggests ‘without apparent external cause’, these spontaneous brain states are likely to emerge from a combination of internal dynamics and context-related inputs from either the external or internal environment (e.g., the presence of a social partner and hunger respectively). Furthermore, internal dynamics can be biased by the physiological state of the body, which is typically signaled by interoceptive afferents to the brain areas of interest under scrutiny.

To detect contextual information in spontaneous LFPs, we use machine learning (ML) techniques in combination with a simple validation procedure. As the application of machine learning to neural data analysis is becoming widespread, we also consider factors that limit the applicability of ML methods in the context of our study.

## Results

### 1. Experimental design

**Fig 1:**
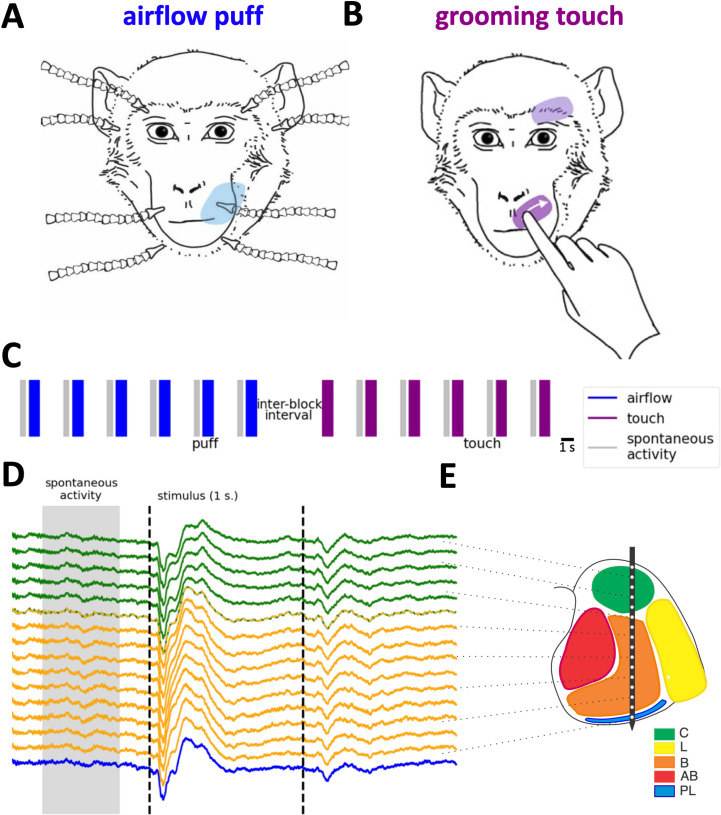
Experimental design. (A) Gentle airflow was delivered by a custom-built system of air nozzles supplied by computer-controlled pressure valves that produced airflows of 10 Pa for a duration of 1 s. The shaded area on the right upper muzzle indicates the spread of skin mechanoceptors activated by the stimulus. (B) Grooming sweeps to the same skin area as in (A) delivered by a trusted human. (C) Time course of the last 6 airflow trials in an airflow block followed by the first 6 grooming trials in the subsequent touch block. Blue and purple vertical lines indicate successive airflow and touch trials, respectively. The width of the line indicates the stimulus duration = 1s. Vertical gray bars indicate the spontaneous activity selected between two stimuli of the same type. Note that there is no selection before the first trial of a new block. (D) Event-related LFP from a sample recording session. Color code of LFP activity corresponds to the estimated location of V-probe contacts in different nuclei of the amygdala. Lines with alternating colors refer to contacts on the boundary of two nuclei (E). Corresponding recording sites in the amygdala. C=central, green; L= lateral, yellow; **B** = basal, orange; AB = accessory basal, red; Pl = paralaminar, blue. Panels (A) and (B) appear as Fig 1 in (Martin et al., 2023) [6] and are used here under the Creative Commons CC-BY 4.0 license.

Three adult male macaques received, in alternating blocks of trials, two types of tactile stimuli: (1) a gentle airflow (not a startling air puff) with a pressure of 10 Pa delivered through airflow nozzles brought to the vicinity of the face, but avoiding the eyes and the nostrils, and (2) gentle grooming sweeps delivered to the same areas of the face by a trusted human partner, who wore an instrumented glove that allowed matching of the contact forces of the airflow and the grooming sweeps (Fig 1). Linear electrode arrays (V-probes) with 32 recording contacts distributed across a span of 6mm from the tip were lowered into the amygdala. The 6mm-span ensured that we recorded LFP from the full dorso-ventral expanse of the amygdala. Unless some of the contacts had excessive noise or were located outside the amygdala (e.g., if probe tip was not advanced to the deepest point in the amygdala) we used the data from all contacts. However, this did not ensure equal sampling of each nucleus because each nucleus has a different location and different size. (A summary of the number of recording electrodes in each nuclei across all sessions is reported in the Methods section.) Nevertheless, on each recording session the V-probes were lowered to different anterior-posterior and medial-lateral coordinates of the amygdala to enable quasi-equal sampling of all regions of the amygdala. The location of the recording electrodes was determined through MRI reconstruction (Fig 1E). The contour of the nuclei (visible or estimated) was drawn on each 0.5mm-thick MRI slice. A grid in the recording chamber allowed targeting of specific nuclei in each slice. For example, a vertical probe insertion targeting the lateral nucleus (in yellow on Fig 1E) would omit the central nucleus, but during an insertion targeting the basal or accessory basal nuclei the uppermost contacts would likely land in the central nucleus (if, for example, the probe in Fig 1E was moved slightly to the left). As the probes were advanced into the brain, we listened and watched the changes in neural activity registered by each contact and verified that these changes occurred at the expected depths of traversing different structures (e.g., cortex, white matter, basal ganglia, anterior commissure, etc.). Based on the location of the top and bottom contact we then drew on the MRI slide corresponding to the anterior-posterior axis of the amygdala the position of each contact relative to the pre-established contour of the nuclei. The maximal margin of error was typically 0.5mm.

**Fig 2:**
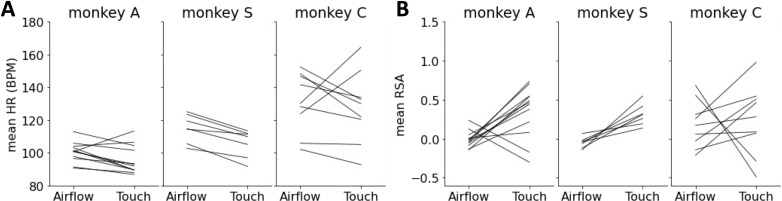
Autonomic state difference in airflow versus grooming blocks. (A) Mean heart rate measurements during airflow and grooming blocks for Monkey A (left), Monkey **S** (center), and Monkey C. (B) Mean RSA strength during airflow and grooming blocks for Monkey A (left), Monkey **S** (center), and Monkey C. The noticeably higher HR in monkey C may be due to generally higher mean and variance of its HR (see [6]).

During grooming blocks, the heart rates of the subjects were significantly reduced compared to airflow blocks, indicating a state of low sympathetic arousal (Fig 2A). Moreover, heart rate variability was increased during grooming, which is a reliable sign of a parasympathetic-dominated physiological state [17] (Fig 2B). Respiratory sinus arrhythmia (RSA), taken over 60s intervals, shows statistically higher strength during grooming than airflow in two of three subjects. (One-sided t-test: Monkey A, p = 0.0018, n=12 sessions; Monkey S, p < 0.001, n=8 sessions; Monkey C, p > 0.05, n=9 sessions.)

Each airflow sequence consists of 11 presentations of the stimulus to pseudo-random locations (10 aimed at the face, one sham). Each presentation of 1s duration is separated by 4s. During a block, the sequence is repeated 10 times for a total of 110 presentations. Each grooming block consists of 20 stimuli, separated by ~4s and repeated 5 times for a total of 100 presentations. A few minutes elapsed between blocks.

### 2. Selection criteria for the relevant segments of spontaneous activity

Spontaneous LFPs were selected from stable time windows of the interstimulus interval (ISI) between two stimuli of the same type. In other words, we did not consider the activity occurring before the first stimulus presentation in each block. The ISI was defined as the period occurring 200ms after stimulus offset and 200ms before stimulus onset. We removed the activity immediately before and after the stimulus delivery to avoid including any neural activity related to the processing of the stimulus. Including any direct information about the stimulus could bias the classifiers to discriminate between the stimulus-evoked responses rather than the spontaneous activity between stimulus delivery.

Additionally, since the grooming touches were delivered by a human experimenter, the ISI between successive grooming stimuli do not have uniform lengths. To ensure uniform time windows for our classifiers, we selected a spontaneous activity window from the ISI for their stable statistical properties. ISI signals in each session were trial averaged and the standard deviation for each timepoint was calculated. Spontaneous LFP for each trial was chosen by inspecting the trial-averaged ISI and determining a time window with low trial-wise variability. See S1 Text for details.

### 3. Machine learning for LFP analysis

Recent years have seen major advances in machine learning (ML) in the biomedical sciences, including cancer diagnosis [18], detection and treatment of Alzheimer’s and Parkinson’s diseases [19,20], and seizure detection [21]. We are particularly motivated by LFP-Net [20], which uses convolutional neural networks to analyze LFP data from human subjects with DBS implants. We implemented our ML-based methodology using two well-known and popular types of classifiers: a convolutional neural network (CNN) based on [20], and a support vector machine (SVM) [22] (see Materials & Methods). The use of two classifiers allows us to check our findings and compare their performance in a practical setting (see S1 Text for more details on ML architectures and comparisons).

Spectral features are often chosen as a reliable feature space for decoding behavior from LFP [23]. To make use of spectral information and at the same time accommodate potential nonstationarity in the data, we use single trial (~500ms) time-frequency plots, or spectrograms, of LFP as inputs to our classifiers. Deep neural networks have rarely been applied to characterize single trial LFP events [24]. Both CNN and SVM leverage statistical methods to nonlinearly transform spontaneous LFP spectrograms and “learn” spatiotemporal patterns (features) that separate airflow from touch in this new feature space. Given that context-related modulation was seen in a fraction of the single units [6], we hypothesize that spontaneous LFP, which records from a larger population of neurons at the mesoscale, may provide a more reliable feature space for successfully decoding context than single-units.

**Fig 3:**
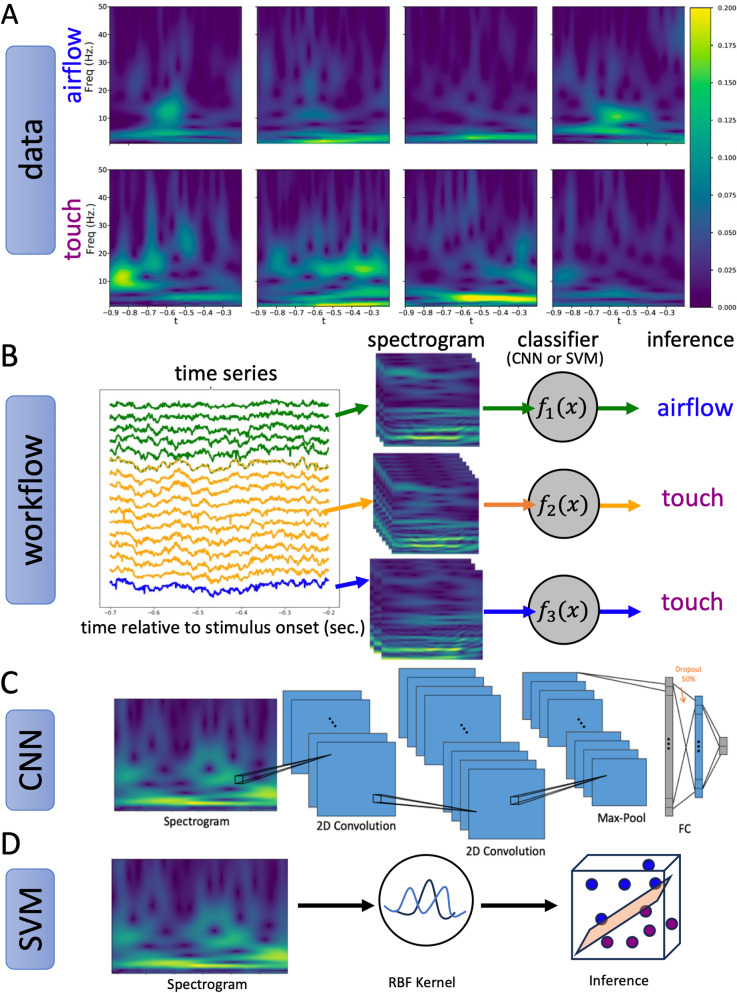
Analysis pipeline. (A) Sample spectrogram images of “airflow” trials (top) and “touch” trials (bottom) used in the training set of a classifier. (B) **Spontaneous** LFP trace from a single trial. Signals recorded from the same nucleus (indicated by the same color) are grouped together. Spectrograms are computed using a complex Morlet wavelet transform and labeled as “airflow” or “touch” depending on block type, then used to train a classifier (CNN or SVM) to discriminate between airflow and touch spectrograms for each nucleus. (C) Architecture of the CNN consisting of two 2D convolutional layers, followed by max-pooling. The outputs are then flattened and fed through two fully connected linear layers and a final 2-node output layer which determines the predicted label of “airflow” or “touch”. (D) Schematic of the SVM classifier consisting of a non-linear embedding using radial basis function (RBF) kernels followed by a linear classifier.

The workflow, the format of the data, and features of the two classifiers used to decode spontaneous activity trials are shown in Fig 3. For each recording session and for each nucleus recorded from on that session, we trained one CNN and one SVM. Spontaneous activity trials were labelled as “airflow” (for neural activity occurring during the spontaneous activity window between two airflow trials in an airflow block) or “touch” (for neural activity occurring between two grooming trials in a grooming block).

For each labelled time window of spontaneous activity, we compute a spectrogram and use the resulting spectrogram-label pairs to form our dataset. Examples of airflow and grooming spectrograms used in training are provided in Fig 3A. To ensure a large enough dataset for each classifier, spectrograms originating from the same anatomical region in a single session were grouped together to form one dataset. That is, the signals from all recording electrodes that are determined to be in the same anatomical region are grouped together. We do this for every session and every nucleus recorded in that session. For each nucleus-session dataset, we trained one instance of a CNN and SVM each (Fig 3B). Each classifier is trained on a random subset of the data (the training set) and a hold-out set (the validation set) is used to measure overfitting. After training converges, the model with the lowest validation loss is chosen as the best model. Lastly, we evaluate the best model performance on a separate hold-out set (the test set).

The architecture of the CNN is shown in Fig 3C and detailed in Methods. The first half of the network consists of two successive 2D convolutional layers, followed by a Max Pool layer. The convolutional layers learn a set of 48 convolutional kernels (in both time and frequency) which identify distinguishing features of the spectrograms in the training set. These features are then flattened and sent to the second half of the network for classification. The second half of the network consists of two fully connected linear layers which perform linear classification on the features learned from the convolutional layers. In Fig 3D, we provide a schematic of the SVM architecture. We train SVM using radial basis functions (RBF) kernel because traditional linear SVM did not discriminate well between “airflow” and “touch”, suggesting that a nonlinear embedding is necessary in our context. These modern machine learning methods are useful for detecting patterns in the training set that are not apparent to the naked eye when looking at example trial spectrograms.

### 4. Context can be reliably decoded from spontaneous LFP in amygdala

**Fig 4:**
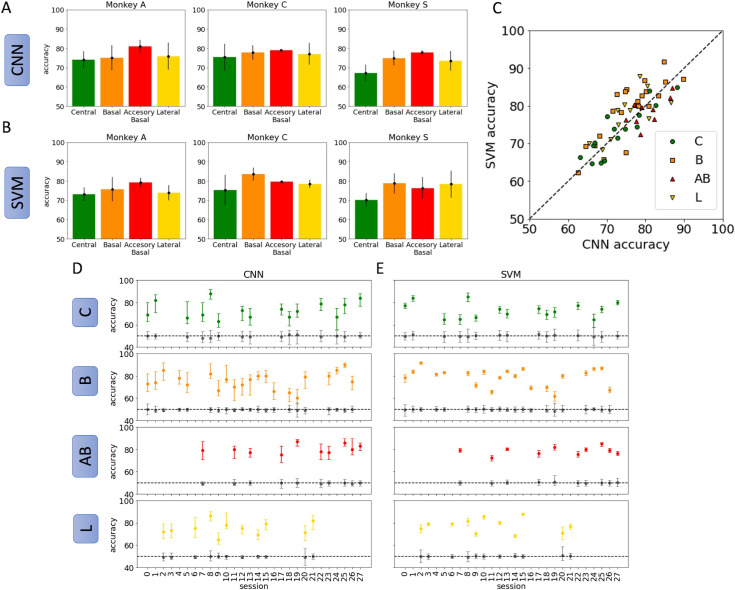
Decoding context reliably from spontaneous LFP spectrograms. Average accuracy was computed over 50 sample CNNs and SVMs. Trials were randomly reassigned to the training, validation, and testing sets for each sample classifier using an 80-10-10 split. (A) CNN classification accuracy for each nucleus, averaged over all sessions for three subjects. (B) SVM classification accuracy for each nucleus, averaged over all sessions for the same subjects.(C) A scatterplot comparing accuracy results for CNN and SVM in each session and nuclei. (D) Accuracy results for all recording sessions using the CNN classifier. The 50% quantile of accuracy is represented by a dot, with vertical bars reporting the 10% and 90% quantiles. Colors indicate the nucleus in which the recording contacts were located. Gray bars indicate the null distribution obtained from bootstrapping. (E) Accuracy results for all recording sessions using an SVM with RBF kernel. Average values and quantiles as in A. Gray bars indicate the null distribution obtained from bootstrapping. C = central, green; **B** = basal, orange; AB = accessory basal, red; L = lateral, yellow.

Results from training distinct classifiers for each recording session and nuclei are shown in Fig 4. For each classifier, we report a summary of the accuracy distribution for a single network computed over 50 instances (more details of the training process are explained in the Methods section). The accuracy for a single instance is calculated as the fraction of correctly labelled spectrograms from the test set; the two labels are equally represented in the test set. This process is repeated 50 times to generate a distribution of 50 accuracy values for a single classifier. We report the 10th, 50th and 90th quantile accuracy for each classifier. There are two classifiers (one CNN and one SVM) for each recording session and each nucleus. Accuracy results using CNNs for all recording sessions across all 3 subjects are shown in Fig 4C Similarly, accuracy results using SVMs for all recording sessions across all 3 subjects are shown in Fig 4D.

Both CNNs and SVM decode context from spontaneous LFP spectrograms reliably. The distribution of accuracies for each session are shown to be consistently above binary chance (50%). Moreover, to ensure that correlated noise in the datasets was not contributing significantly to network accuracy, we sampled from a pseudo-null distribution for each classifier using a bootstrap method. The null distribution is calculated by first shuffling the labels of the training set so that spectrograms are randomly assigned to the “airflow” and “grooming” task equally. The training process is repeated as usual. Using this procedure, we obtain a “null distribution” of accuracies arising from the model performance on true-labelled test set. This gives an estimation for the likelihood of obtaining accuracies better than 50% in a given dataset due to correlated noise. For each recording session and across nuclei, there is no overlap with the null distribution for either classifier. This gives confidence that the classifiers are not decoding context due to chance. The variability in performance across repeated training for each nucleus can be accounted for, in part, by the number of recording contacts present in each

nucleus during a recording session. As expected, the accuracy of both CNN and SVM classifiers is positively correlated on the number of contacts present in that region during a recording session (see S1 Text).

### 5. Discriminatory power for context is not nucleus-specific

Next, we determined whether contextual encoding was localized to a particular nucleus within the amygdala. We hypothesized that activity recorded from the basal and accessary basal nuclei would be more reliable for decoding context-related information given that they receive more direct inputs from the prefrontal cortex. Contrary to our expectations we found no difference in decoding accuracy across the nuclei with either classifier type (Fig 4A and 4B). This is confirmed by applying the Kruskal-Wallis H-test (or one-way ANOVA on ranks) across the four conditions: we failed to reject the null hypothesis with p > 0.1 in all three subjects. These results suggest that the context-related signals in the spectrograms of spontaneous activity do not depend on the hypothesized inputs from the prefrontal cortex, rather, contextual information encoded in spontaneous activity arises from inputs that are distributed quasi-equally across the nuclei of the amygdala.

## Discussion

In neurophysiology, “baseline” refers to the spontaneous ongoing activity of neurons in the absence of the organism’s engagement with external stimuli or task variables. External stimuli and cognitive processes shift the brain away from baseline toward task-specific or stimulus-specific functional states. However, at the cessation of the external stimulus or the completion of the cognitive process, the brain is expected to return to the same baseline state or spontaneous activity. Here we show that this is not always the case; when the context is different the spontaneous activity is also different. Although few studies explored the information contained in spontaneous activity, the discovery of intrinsic dynamics of the brain, such as memory replay [25], the default mode network (e.g., [26]), and off-task cognitive process [27], raised the possibility that this activity carries relevant and decodable information [28]. In our study, the most parsimonious account for the persistence of the contextual information in the spontaneous activity during the inter-trial period is that context operates on a significantly longer time scale than the individual stimuli and this time scale spans the entire block. The context (social and non-social) is present throughout the experiment, akin to a background, and the individual stimuli are processed in relation to this context.

As context is often signaled to the primate amygdala by the prefrontal cortex [29,30], it is expected that the nuclei that receive direct prefrontal inputs would show the strongest context-related activity. Specifically, the basal and accessory basal nuclei of the amygdala receive monosynaptic inputs from multiple prefrontal areas whereas the lateral and the central nuclei are connected to the prefrontal cortex through multi-synaptic pathways [31–34]. Contrary to this prediction, both CNN and SVM decoded with similar accuracy context-related signals from the LFPs in all the nuclei of the amygdala. It is unlikely, therefore, that the spectrograms of spontaneous activity are shaped by direct synaptic currents transmitted from prefrontal areas to select nuclei of the amygdala. It is more likely that a broader, global phenomenon, such as the autonomic state of the animal, is signaling context to the amygdala. Indeed, we report significantly different autonomic states during the airflow and the touch blocks (Fig 2). During the airflow blocks, the monkeys are by themselves in a booth, and while they receive innocuous airflow stimuli directed at their face, they are alert, attentive, and highly responsive to external stimuli. During the touch blocks, when a bonded and trusted human grooms their face, the monkeys close their eyes and relax their facial musculature. The groomer’s presence is marked by reductions in heart rate and increases in heart rate variability that indicate lower sympathetic tone and higher parasympathetic tone [17,35]. The autonomic state of the body is transmitted to multiple areas of the brain through interoceptive pathways [36–38]. It is possible, therefore, that the spontaneous activity across all nuclei of the amygdala is shaped by interoceptive inputs that were widely different during the airflow and touch blocks. Similar modulation of the spontaneous activity by interceptive inputs have been documented in mice [39]. Whether LFPs in different nuclei of the amygdala carry different or similar signals, it remains unclear how context-related or interoception-related features of LFPs might contribute to decoding accuracy.

### Comparison with previous work

In this study the LFPs showed lower decoding accuracy compared to single unit population activity during the same periods of spontaneous activity [6]. This may be related to the short periods examined (approximately 500 ms, but in some cases as short as 400 ms) and the low number of trials (about 400 trials for training) available for session and subject specific analysis. In the single unit approach, stable cells identified across all recording sessions and subjects (a total 237 units) were used to train a single SVM. These cells were randomly sampled with replacement to train the SVM and it was determined that a population of 127 single units were predictive of context at the 95% confidence level. This population size of single units is far greater than the number of stable cells recorded in an individual session and makes no distinction between nuclei. Given that our LFP approach is nucleus-, session-, and even subject-specific, it is to be expected that decoding accuracy with LFP is lower than with single unit activity.

### ML methodology

In this paper, we used a simple framework based on training and cross-validating ML classifiers. A positive result, i.e., accurate classification of LFP samples, is interpreted as evidence that the LFP encodes the contextual information we seek. However, we caution that the converse need not be true: there are a number of reasons why the classifier may not accurately discriminate between airflow and touch; had we found a negative result, it need not mean contextual information was absent. The ML classifier may have been unable to “learn” a good representation from the given data due to constraints imposed by its architecture and/or training protocol; after all, our CNN follows the original LFP-Net design very closely, and it may be that a neural network model with more parameters would be able to “discover” better representations. Or, the dataset may have been too small, the measurements too noisy, or there may have been other limitations imposed by the experimental design. Indeed, we showed that the same ML classifiers (CNN and SVM) trained on raw time series data could not discriminate airflow and touch stimuli accurately, but classifiers trained on spectrograms can do so rather accurately. This reinforces the common understanding that representation of data can significantly impact the efficacy of ML classifiers, and the standard ML techniques used in this paper do not seem to be able to “discover” time-frequency representations on their own [40].

A second example illustrating the limitation of our methodology (detailed in Fig G in S1 Text) explores the question of whether contextual information is predominantly carried by rhythmic activity in specific frequency bands. There, as a first step toward studying this question, we restricted our attention to those frequency bands showing the greatest change in spike-triggered average power under the two stimulus classes. We found that the classification accuracy CNN degraded significantly when restricted to this narrow band, suggesting that CNN require broad-band information for accurate classification. But a second test statistic, specifically the area under the receiver operating characteristic curve, did not indicate a difference between CNNs trained on broad-band and band-limited data. These outcomes suggest that the question of whether contextual information is encoded in specific measurements can be subtle: it depends not only on the data at hand and on the classifier architecture, but also on the choice of task and test statistic.

The ML approach we took has other limitations: like many ML methods, the methods used here are opaque and do not directly tell us which neurobiological features contribute the most to classification accuracy. Furthermore, our classifiers are not generalizable: they are trained on data obtained from different subjects and sessions when the linear probes recorded neural activity from different nuclear subdivisions of the amygdala, and our classifiers do *not* generalize when applied to data obtained from other sessions (see S1 Text). This was expected based on previous work that mapped dissociable functions to different mesoscale subregions of the amygdala [41]. For the purpose of this paper, our (non-generalizable) approach was sufficient to conclude that contextual information is present in spontaneous LFP.

It remains to determine which features of LFPs were used by our classifiers to achieve their performance. We expect future work focusing on baseline activity in the amygdala will shed light on how this often-ignored feature of brain activity holds specific information about context, interoception, and other aspects of brain states. We suspect simpler, more biophysically-interpretable classifiers that disentangle biological sources of variability from measurement-specific variability (e.g., those related to probe placement, etc.,) may improve overall generalizability and may be more robust to variability across subjects and small changes in probe placement. Such ML models may also enable feature identification. In a different direction, more general classifier architectures (e.g., foundation models) may improve automatic discovery of effective representations. In any case, if we are able to isolate those features of LFP used by our ML classifiers to achieve their performance, then ML methods – in addition to being effective information detectors – would be useful for generating new hypotheses and may directly contribute to a clearer picture of contextual information coding in the amygdala and elsewhere.

### Concluding remarks

Despite the limitations mentioned above, the spectrogram of short segments of LFPs recorded during periods of spontaneous neural activity were sufficiently different to allow two different classifiers to discriminate the social context in which the two types of tactile stimulation took place. While it is unclear which features of the LFPs carry the contextual signal, it is apparent that this signal is retained in the spontaneous activity of mesoscale network in the amygdala and remains unchanged for the duration of the block. These identifiable, spontaneous states, specific for context, were similar across the component nuclei of the amygdala. In contrast, our previous study on the stimulus-evoked LFPs in the amygdala showed remarkable differences between the component nuclei, attesting to the presence of functional subnetworks with highly differentiable responses to visual, auditory, and tactile stimuli [41]. It is remarkable that despite such inter-nuclear differences, the networks that give rise to context-specific inter-trial LFPs converge on two patterns and each pattern is decodable from all nuclei. We surmise, that at least in this case, interoceptive afferents that signal the physiological state of the body, which was different between the grooming and the airflow blocks, might override the anatomical and functional distinctions between the nuclei. It is possible that sustained internal states that instantiate the social context may predispose the amygdala to evaluate differently the salience, valence, and behavioral significance of external stimuli.

## Materials and methods

### Experiment

We did not conduct any new experiments for this paper. All data involving live subjects were collected as part of an earlier study [6]; see the Methods section of that paper for details. In terms of data provenance and localization of LFP sources to different amygdala nuclei we used the following approach. In monkey A, we used initially probes with 16 channels distributed over a shaft length of 6mm. The distance between the contact was 400 microns. Given that these probes were lowered into the amygdala along its vertical axis, and along this axis the central nucleus is only 1.5-2mm in diameter whereas the basal nucleus is 4 mm, we collected LFPs from two contacts in the central and up to 10 contacts in the basal nucleus. Naturally as we sampled the more anterior and posterior “slices” of the amygdala these numbers changed because the more anterior slices do not have a central nucleus whereas the more posterior slices have smaller basolateral nuclei. Each recording session was different not only because we were recording from different anterior-posterior locations in the amygdala but also because the probes were not lowered every day to the same depth. In monkeys S and C we graduated to probes with 32 contacts that doubled the yield but retained the unequal number of recording sites from each nucleus. Finally in monkey C we recorded simultaneously from the left and right amygdala, further increasing the yield but retaining the unequal sampling. The location of each contact was established based on two sources of information, as follows: (1) On the MRI-based atlas constructed for each monkey, we drew on each 1mm slice the estimated contour of the nuclei (these contours were rarely visible, but we developed a method of estimation that has been validated by post-mortem histology). (2) As the probes were advanced into the brain, we listened and watched the changes in neural activity registered by each contact and verified that these changes occurred at the expected depths of traversing different structures (e.g., cortex, white matter, basal ganglia, anterior commissure, etc.). Based on the location of the top and bottom contact we then drew on the MRI slide the position of each contact relative to the pre-established contour of the nuclei. The maximal margin of error was typically 500 microns, i.e., 2 contacts mis-assigned to the nuclei.

### Summary of recording electrodes sampling for each nuclei

For each recording session, the V-probes were lowered to different anterior-posterior and medial-lateral coordinates of the amygdala. Given the difference in positioning and relative size of the nuclei, the number of recording electrodes sampling each nuclei is variable across sessions. We present a summary of the number of recording electrodes across all sessions in Table 1 below.

**Table 1:** Summary of recording electrode distribution across sessions.

Nucleus	Min	20%	50%	80%	Max
**Accessory Basal**	7	9	14	22	24
**Basal**	1	3	12	21	32
**Central**	1	1	4	6	12
**Lateral**	2	5	20	26	32

### Materials

All models and data analysis were run with Python using popular open-source packages like *Pytorch-1.11.0, numpy-1.22.4, scipy-1.12.0, and scikit-learn-1.1.1.* We have released all relevant source code at https://github.com/anaucoin/Aucoin-ML-LFP-2024. Data available upon request.

### Heartrate and respiratory sinus arrhythmia

Instantaneous heartrate values were computed from the inverse of the duration between two heartbeat times (IBI). Values above 240 BPM or below 40 BPM (IBI below 250ms and above 1500ms) were removed. All noise and movement artifacts identified were also removed. Heartbeat values were interpolated to a 1ms timescale using a modified Akima cubic Hermite polynomial.

Heartrate variability [17,42] was calculated using a spectral density estimation method. Spectral power density was computed from the cleaned heartbeat times using a multitaper method in sliding windows of 60 seconds and an overlap of 3s. A total of 7 Slepian tapers were used for smoothing. The set of k Slepian tapers are the first k eigenvectors found by solving an eigenvalue problem ordered by the eigenvalues such that the 0^th^ taper is the eigenvector associated with the largest eigenvalue [43]. For all sequences $x_{n}\in$ $l^{2}$ of length N, the sequences with maximal energy concentration on the interval $\left[-W,W\right]$ are the eigenvectors $g_{k}\left(n\right)$ which satisfy


$$\begin{array}{c} \sum_{m=0}^{N-1}\frac{sin\left(2\pi W\left(n-m\right)\right)}{\pi \left(n-m\right)}g_{k}\left(m\right)=\lambda_{k}\left(N,W\right)g_{k}\left(n\right),n,k=0,1,\ldots ,N-1. \end{array}$$ (1)

Here, *W* is the half bandwidth and is chosen to be less than the Nyquist frequency $\left|W\right|<\frac{1}{2\Delta t}$. We chose W=.07 for our context for.07 Hz smoothing. The multitaper method computes the power spectra in each time window as the average of *k* modified periodograms

such that


$$\begin{array}{c} S_{k}\left(f\right)=\Delta t\left|\sum_{n=0}^{N-1}g_{k}\left(n\right)x\left(n\right)e^{-j2\pi fn\Delta t}\right| \end{array}$$ (2)

$$S\left(f\right)=\frac{1}{k}\sum_{i=1}^{k}S_{i}\left(f\right).$$

For each time window $b_{i}$, the power spectra $S^{b_{i}}\left(f\right)$ were normalized to have unit area between 0.25 Hz and 0.5 Hz, corresponding to respiratory rates of 15 and 30 breaths per minute. We define respiratory strength in each time window as the average power at the peak  ±  half bandwidth for peaks occurring between 0.25 Hz and 0.5 Hz. In time windows with no peaks, the average power across the entire 0.25 to 0.5 Hz window was used. This sequence of respiratory strengths is a measure of RSA in each time window denoted by $P_{rsa}\left(b_{i}\right)$. RSA strength is then normalized using the median power across all time windows $\mu =median\left(P_{rsa}\left(b_{i}\right)\right)$ to compare. Thus, RSA strength is given by


$$\begin{array}{c} {\tilde{\text{P}}}_{rsa}\left(b_{i}\right)=\frac{P_{rsa}\left(b_{i}\right)-\mu }{\mu }. \end{array}$$ (3)

### Spectrogram computation

To compute trial spectrograms, we use a Continuous Wavelet Transform (CWT) method [44]. Unlike traditional spectral methods like Fast-Fourier Transform (FFT), which collapse time, CWT provides a trade-off between spectral and temporal resolution. CWT is a natural choice as we do not expect trial LFPs to be stationary. For a given signal $x\left(t\right)$, the CWT $\hat{x}\left(f,t\right)$ is defined as


$$\begin{array}{c} \hat{x}\left(f,t\right)=\int_{-\infty }^{\infty }x\left(\tau \right)\frac{1}{\sqrt{a}}\bar{\psi }\left(\frac{\tau -t}{a}\right)d\tau ,a=\frac{2F_{s}}{f} \end{array}$$ (4)

where $\bar{\psi }\left(t\right)$ denotes the complex conjugate of the wavelet function $\psi \left(t\right)$, and $F_{s}$ is the sampling rate of the given input signal $x\left(t\right)$.

For each trial of spontaneous LFP, our choice of wavelet function $\psi \left(t\right)$ is a Complex Morlet Wavelet


$$\begin{array}{c} \psi \left(t\right)=\frac{1}{\sqrt{\pi f_{b}}}exp\left(\frac{-t^{2}}{f_{b}}\right)exp\left(j2\pi f_{0}t\right) \end{array}$$ (5)

where $f_{0}$ is the central frequency and $f_{b}$ is the bandwidth. All spectrogram images were computed using the *scipy.signal.cwt* function with ‘Morlet2’ wavelet and a standard central frequency of 5 for a good balance of temporal and frequency resolution. To reduce model training computation time, we limited the input image size by considering only frequencies in the range of 1–50 Hz for each spectrogram. Similar networks were trained with frequencies up to 100Hz and showed no significant increase to model accuracy.

### Convolutional neural network

Convolutional Neural Networks (CNNs) are deep multi-layer networks widely used in computer vision and image classification tasks [45]. The simple CNN architecture used in our paper can be thought of in two parts: (1) a convolution operator and (2) a linear classifier. The layers in the convolutional part of a CNN typically include three operations:

1. Convolution
2. Non-linear activation
3. Pooling

Though not all operations need be in every “layer” of the network. The convolutional layer involves convolving an input image *x* with a collection of K kernels of size s×s. *s* is typically chosen to be small to preserve locality. In our network, *s* =3. The kernels are learned by the network during training and output a collection of K local features. These features are then passed through a non-linear activation function (typically ReLU) and then through a pooling operation (such as Max or Average Pooling). The non-linear activation can be thought of as thresholding and increasing the receptive field in a biological sense, and the pooling operation serves to further reduce dimensionality. These steps can be repeated to increase the depth and complexity of the network. The result of these successive operations is a collection of features extracted from each input image. These features are then flattened into a single vector and used as input into the linear classifier part of the CNN. This second half of the network consists of a series of fully connected linear layers, which compose a linear map that transforms the flattened features into an output vector *y* of length $C_{n}$ the number of classes. Each $y_{C_{i}}$ describes the probability that the input image *x* belongs to class $C_{i}$. The weights and biases of the linear map are optimized during training.

The exact model architecture and parameters used in this analysis, can be found in Table 2. For each recording session, and for each nucleus recorded in that session, we trained a CNN to classify spectrograms of the spontaneous LFP signals as “airflow” or “touch”. The spontaneous spectrograms for all channels in a particular anatomical region were used as inputs into the network for the training, validation, and testing stages. Bootstrapping (random sampling with replacement) was used to sample the “touch” images so that there was an equal number of “touch” and “airflow” images in the dataset. The data was then split into training, validation, and testing subsets with an 80-10-10 split. An equal class representation was ensured in each split. Each input image was normalized using the “MinMaxScaler” from *scikitlearn* package fit only on the training set to prevent erroneously giving the network information about the entire dataset. The network parameters were updated through back propagation using the Adam optimizer and a cross-entropy loss function was used.

**Table 2:** Summary of model architecture. Note that ${\text{L}}_{\text{i}}$ and ${\text{W}}_{\text{i}}$ will vary based on frequency bands analyzed and length of input signal.

*Type of Layer*	*Size of output feature map*	*Kernel size*	*Stride size*
Convolution + ReLU	$L_{1}\times W_{1}\times 24$	3×3	1×1
Batch Normalization	$L_{1}\times W_{1}\times 24$	N/A	N/A
Convolution + ReLU	$L_{2}\times W_{2}\times 48$	3×3	1×1
Max Pool	$L_{3}\times W_{3}\times 48$	2×2	1×1
Batch Normalization	$L_{3}\times W_{3}\times 48$	N/A	N/A
Fully Connected	64	N/A	N/A
Dropout	64	N/A	N/A
Fully Connected	2	N/A	N/A

The data was fed into the model in mini batches of size 20 and trained for 40 epochs. This means that the model saw 20 spectrogram images before updating the model parameters and saw all spectrogram images a total of 40 times. During training, the model was shown spectrogram images from the validation set to track over-fitting of the model. If the loss of the model decreased on the validation set, the model parameters were saved as the current best instance of the model. Once all 40 epochs were complete, the best model (the model with the lowest validation loss), was used in the testing stage of the model to obtain the AUROC metric. To analyze the accuracy and robustness of each model, we repeated this procedure 50 times: we reinitialized the model, randomly assigned spectrograms to the training, testing, and validation sets, trained the new model and recorded the accuracy on the test set. The accuracy reported for each model is the average percentage of correctly classified images over 50 model initializations. The spread of accuracy values for each distinct classifier is displayed by the 10^th^ and 90^th^ quantiles.

### Support vector machine

Support Vector Machines (SVM) are a popular supervised learning method for classification and regression [46]. They have been shown to be effective even in high dimensional settings and settings where the number of dimensions is much higher than the number of samples, such as classification of gene expression data [47] and image segmentation at the pixel level in microarray images [48]. SVMs construct a high-dimensional hyperplane, called the decision boundary, to separate training data by their labeled class. Consider a training set of pairs $\left(x_{i},y_{i}\right)$ where $y_{i}$ is either +1 or -1 denoting which class $x_{i}$ belongs to. If the training data is linearly separable, we can characterize a maximum-margin hyperplane as a vector satisfying $w^{T}x+b=0$. Two paralell hyperplanes (1) $w^{T}x+b=1$ and (2) $w^{T}x+b=-1$ can then be found to determine the decision boundary. If $x_{i}$ lies above (1), it is labelled as belonging to the +1 class. If $x_{i}$ lies below (2), it is labelled as belonging to the -1 class. The maximum margin hyperplane is chosen to maximize the distance between the decision boundary and nearest data points. In the linear classifier case, this is equivalent to solving the following optimization problem to find the optimal model parameters:


$$\begin{array}{c} w_{fitted}=\underset{w}{argmin}Loss\left(w\text{|}data\right) \end{array}$$ (6)

In the case where a linear decision boundary is insufficient, the linear classifier can be made nonlinear through a non-linear kernel function. The kernel functions embed the data $x_{i}$ into a new vector space called the feature space. This procedure is sometimes called the “kernel trick”. The new optimization problem replaces the data in (6) with a non-linear transformation of the data


$$\begin{array}{c} w_{fitted}=\underset{w}{argmin}Loss\left(w\text{|}\phi (data\right)) \end{array}$$ (7)

where *ϕ* is the choice of kernel function. In practice, the dual formulation of this optimization problem is used to avoid explicitly mapping the data points into the feature space. This helps to better scale the computational efficiency and memory usage, especially when the number of features in the data is high.

To train the SVM, we used svm.LinearSVC and svm.SVC from the popular *scikitlearn* package. We trained a non-linear classifier with radial basis kernel functions using the dual form for efficiency. The input data into the SVM are the flattened spectrograms labelled as “airflow” or “touch”. The dimension of the input data is T×F where T is the number of time points and F is the length of the frequency space discretization used. F and T will vary on the frequency bands of interest and the length of spontaneous LFP for that session. Training, validation, and testing of the SVMs were the same as in training of CNN.

## Supporting information

S1 Text Fig A. Selecting stable trials of spontaneous activity. An example of the spontaneous LFP selection criteria for a single recording session. The solid blue line is the trial-average of the signals during each ISI. The vertical green line is stimuli onset. The light blue shading is 2 st. dev. of the mean. The gray box is the stable time window chosen as the spontaneous activity window. Fig B. Decoding context fails with linear SVM. Average accuracy was computed over 50 sample linear SVMs. Trials were randomly reassigned to the training, validation, and testing sets for each sample classifier using an 80-10-10 split. Accuracy results for all recording sessions using the CNN classifier. The 50% quantile of accuracy is represented by a dot, with vertical bars reporting the 10% and 90% quantiles. Colors indicate the nucleus in which the recording contacts were located. Gray bars indicate the null distribution obtained from bootstrapping. Fig C. Decoding accuracy is a function of data availability (number of recording electrodes). Classification accuracy for one sample of CNN (left) and SVM (right). Each dot represents the accuracy of a single classifier trained on data from one session and nucleus. Fig D. Computational costs of CNN and SVM. The computational time for training a single classifier for CNN (left) and SVM (right) as a function of the number of recording electrodes (which most influences training dataset size). Each dot represents a single realization of the classifier. Fig E. Comparison of SVM using raw timeseries data over spectrogram. SVM classification accuracy for each nucleus, averaged over all sessions for the same subjects (Monkey A (left), Monkey C (middle), Monkey S (right)). More saturated colors indicate the SVM accuracy using raw time series trials. SVM decoding accuracy using trial spectrograms (reported in the main text) is shown in more transparent color for comparison. Fig F. Validation loss scales poorly for data from other sessions. Training (blue) and validation loss (orange) for data from the same session. Using a validation set from a different data set shows that although correlated, the loss values do not generalize as well on data from alternate sessions (green). Fig G. Classifier accuracy on band-limited data. (A) Left: Mean Spike-Triggered Average (mSTA) traces for “airflow” (blue) and “touch” (purple) computed for ±80 ms relative to spikes occurring during baseline. mSTA is computed by averaging STAs over all cells in the same nucleus. The number of visible lines corresponds to the simultaneously recorded stable cells in each nucleus used to compute mSTA. Pale lines are single STA traces and dark lines are the mean STA. Right: Comparison of average power spectra of STA traces for airflow and grooming blocks. The two spectra show differences in the 10–17 Hz (yellow) and 17–25 Hz (blue) bars. (B) A trial spectrogram illustrating the power in the 10–17Hz (yellow) and 17–25Hz (blue) frequency bands. (C) Accuracy of CNNs trained on spectrograms restricted to only 10-17 Hz (yellow), 17–25Hz (blue) and 10–25 Hz (green) bands. Plots are organized by nucleus (rows: Central, Basal, Accessory Basal, Lateral) and recording session (columns), with number of recording contacts in each nucleus displayed in the top left corner. The horizontal black dotted line represents the mean accuracy of the network trained on the full spectrogram. The horizontal blue line represents theoretical chance at 50%. The gray bars correspond to 1 and 2 standard deviations about the mean. Fig H. AUC show no distinct difference between network discriminability using broadband versus band-limited data. AUROC of CNNs trained on spectrograms restricted to only 10–17 Hz (yellow), 17–25Hz (blue) and 10–25 Hz (green) bands. Plots are organized by nucleus (rows: Central, Basal, Accessory Basal, Lateral) and recording session (columns), with number of recording contacts in each nucleus displayed in the top left corner. The horizontal black dotted line represents the mean accuracy of the network trained on the full spectrogram. The horizontal blue line represents theoretical chance at 50%. The gray bars correspond to 1 and 2 standard deviations about the mean. (DOCX)
