# Supplementary material for: Detection of latent brain states from spontaneous neural activity in the amygdala
Source: PLoS Comput Biol. 2025 Feb 13;21(2):e1012247. doi: 10.1371/journal.pcbi.1012247 (PMC11844889; doi:10.1371/journal.pcbi.1012247)
Supplement: S1 Text — (DOCX) [file pcbi.1012247.s001.docx]

**Supporting Information for Detection of Latent Brain States from Spontaneous Neural Activity in the Amygdala**

- **Authors:** Alexa Aucoin^1^, Kevin K. Lin^1,2,*^, Katalin M. Gothard^3^
- **Affiliations:** ^1^Program in Applied Mathematics, University of Arizona, Tucson, Arizona, United States of America, ^2^Department of Mathematics, University of Arizona, Tucson, Arizona, United States of America, ^3^Department of Physiology, University of Arizona, Tucson, Arizona, United States of America

[*klin@math.arizona.edu](mailto:*klin@math.arizona.edu)

**Spontaneous activity selection criteria**

​​Spontaneous LFP was selected from a stable time window of the interstimulus interval (ISI) between two stimuli of the same type. The ISI was defined as the period occurring 200ms after stimulus offset and 200ms before stimulus onset. For each recording session, all ISI signals were trial averaged and the standard deviation for each timepoint was calculated. Spontaneous LFP for each trial was chosen by inspecting the trial-averaged ISI and determining a time window with low trial-wise variability.

Fig A. **Selecting stable trials of spontaneous activity.**  An example of the spontaneous LFP selection criteria for a single recording session. The solid blue line is the trial-average of the signals during each ISI. The vertical green line is stimuli onset. The light blue shading is 2 st. dev. of the mean. The gray box is the stable time window chosen as the spontaneous activity window.

**Linear SVM fails to discriminate context reliably**

Spectrogram classification failed on SVM without using the kernel trick. This is likely because the time-frequency features are highly correlated and require a non-linear mapping to improve separability. Results from the linear SVM classification are shown in **Fig B**. Mean accuracy across nuclei and sessions rarely exceeds 60% and often is not statistically different from the mean of the null distribution found through bootstrapping. Overall, the linear SVM classification is less reliable than performance from both SVM with kernel trick and the CNN, both of which leverage non-linear transformations of the data before performing classification, indicating that the non-linear embedding is necessary for classification.

Fig B. **Decoding context fails with linear SVM.** Average accuracy was computed over 50 sample linear SVMs. Trials were randomly reassigned to the training, validation, and testing sets for each sample classifier using an 80-10-10 split. Accuracy results for all recording sessions using the CNN classifier. The 50% quantile of accuracy is represented by a dot, with vertical bars reporting the 10% and 90% quantiles. Colors indicate the nucleus in which the recording contacts were located. Gray bars indicate the null distribution obtained from bootstrapping.

**Classification accuracy depends on dataset size**

Variability across recording sessions and nuclei is due, in part, to the number of recording electrodes present during recording sessions, which determines the total amount of data. Machine learning methods are notoriously data-hungry, and typically require a large amount of data to adequately learn a particular task. **Fig C** show the classification accuracy as a function of the number of recording electrodes in a particular nucleus during a recording session. Results for all three subjects are shown. For both CNN and SVM, the accuracy of days with a single recording electrode is below 60% but quickly improves with the addition of more data. Both classifiers also show performance generally plateauing between 70-85% which can be achieved with 5 or more contacts present. This exploration explains why performance of the classifier on the central nucleus is (slightly) lower than other nuclei across subject. The relative size of the central amygdala compared to other nucleus is much smaller, limiting the total amount of electrodes that can be present in central amygdala in any given session. Nevertheless, we still see both SVM and CNN classifiers performing better than chance when data from more than one recording electrode is available.

Fig C. **Decoding accuracy is a function of data availability (number of recording electrodes).** Classification accuracy for one sample of CNN (left) and SVM (right). Each dot represents the accuracy of a single classifier trained on data from one session and nucleus.

**Computational cost of CNN and SVM**


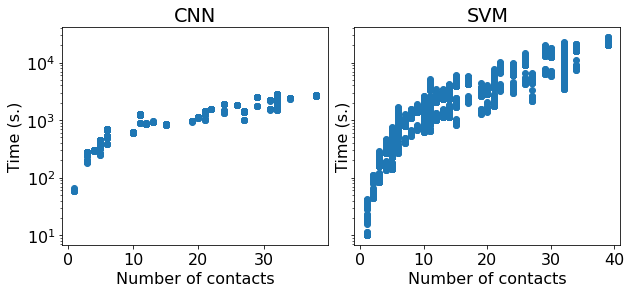


Fig D. **Computational costs of CNN and SVM.** The computational time for training a single classifier for CNN (left) and SVM (right) as a function of the number of recording electrodes (which most influences training dataset size). Each dot represents a single realization of the classifier.

Accuracy results in the main body of the text suggest that although SVM and CNN perform the same on average, the SVM classification exhibits less variability across the 50 realizations (evidenced by the narrower confidence intervals). Readers may be tempted to assume that SVM implementation should therefore be preferred over CNN. However, the choice in classifier may be better decided by the amount of data available. **Fig D** shows the computational cost of implementing both CNN and SVM as a function of the number of recording electrodes. As mentioned above, the number of recording electrodes will strongly influence the amount of data in the training set. For small amount of data (<4 electrodes), SVM is more efficient . However, overall, SVM scale quadratically with the number of electrodes (as expected) while the CNN scales linearly. For moderate and large amounts of data, the CNN is more efficient.

**Classification performance decreases dramatically using raw time-series alone**

The methodology presented in the main text uses trial spectrograms as the feature space for decoding. As a first step in our analysis, we tried traditional classification methods, like SVM, using the raw time series data. Though these methods were able to discriminate between airflow and grooming contexts, the accuracy results were little better than chance. **Fig E** shows the accuracy of SVM decoding using the raw time series trials compared to spectrograms. Decoding accuracy noticeably improves across all subjects and nuclei when using time-frequency spectrograms (Monkey A: 10% increase, Monkey C: 18%, Monkey S: 18%)

Fig E. **Comparison of SVM using raw timeseries data over spectrogram.** SVM classification accuracy for each nucleus, averaged over all sessions for the same subjects (Monkey A (left), Monkey C (middle), Monkey S (right)). More saturated colors indicate the SVM accuracy using raw time series trials. SVM decoding accuracy using trial spectrograms (reported in the main text) is shown in more transparent color for comparison.

**CNN does not generalize well on data from other sessions**

In the Discussion (see main text), we emphasized that our classifiers are not generalizable. Recall that each classifier is trained on data obtained from different subject and sessions when the linear probes recorded neural activity from different nuclear subdivisions of the amygdala. In **Fig F,** we show an example of how the loss values evolve during training when computed using the training set, the validation set, and an alternate data set consisting of spontaneous LFP spectrograms recorded during a different session. The training and validation datasets come from the same recording session with 80% of the data randomly selected for training and 10% for validation. (The remaining 10% makes up a holdout set used for model evaluation and are not presented in the figure.) For the alternative set, we randomly select 10% of the data in a different session (from the same nucleus). In each epoch, we compute the cross entropy loss of the training, validation, and alternative dataset. The loss is computed for each minibatch (consisting of 20 spectrograms) and we report the sum total of the loss over all minibatches. Since the datasets are of varying sizes, we scale each total loss by the total number of minibatches in each dataset for comparison. Though the loss values seem correlated, the classifier clearly does not generalize effectively as evidenced by the discrepancy in loss values of the data set from another session compared to the training and validation sets.

Fig F. **Validation loss scales poorly for data from other sessions.** Training (blue) and validation loss (orange) for data from the same session. Using a validation set from a different data set shows that although correlated, the loss values do not generalize as well on data from alternate sessions (green).

**Classifier trained on band-limited data shows limitations of current study**

A natural question is whether contextual information is carried predominantly by rhythmic activity in specific frequency band(s). As (1) we are unaware of prior studies in the primate amygdala that would bias our expectations for any frequency band, and (2) single units from our dataset showed context-related activity, we looked for consistent differences in power spectrum of the spike triggered average (STA) LFPs between the touch and airflow blocks. The STA gives the conditional expectation of the local field potential given the occurrence of a spike during the spontaneous activity window, filtering out activity unrelated to spikes in the amygdala and providing a representative time series whose power spectra can be used to identify key frequency bands involved in context encoding.

In more detail, for each session and each recording electrode, STA were computed by selecting a window of $\pm$80ms around those spikes occurring during the spontaneous activity period. The STA was then computed as the average over all windows of the same contextual type (airflow or grooming). Power spectrum of the STA were computed using Welch’s method with Hanning tapers, a segment length of 1ms and 50% overlap. Mean STA (mSTA) and average STA spectrums were taken as the average STA and average spectrum over all electrodes in the same nucleus We also remove the 1/f scaling from the spectrum using the FOOOF method (Donoghue et a. 2020). From the power spectrum, we identified relevant frequency bands as the frequency intervals which showed maximal differences in power under the airflow and touch conditions. We found a different pattern in each monkey. Moreover, the short duration of some data segments (400ms) suggests that our data may not be informative for the lowest frequency bands (e.g., theta in monkeys is between 2-5Hz). Nevertheless, we selected the monkey whose recording’s power spectrum showed the largest difference between spontaneous activity of airflow and touch blocks. In monkey A we identified two prominent differences in power between airflow and touch spontaneous activity trials, 10-17Hz and 17-25Hz. The lower band having more power during the spontaneous activity period between the “touch” trials across multiple (but not all) sessions and the 17-25Hz band having more power during the spontaneous activity period between “airflow” trials consistently across recording sessions (**Fig GA**). These data showed that all frequencies contribute to the CNN’s ability to decode context from the spontaneous LFPs (**Fig GC**). However, when we compared the Area Under the Receiver Operating characteristic Curve (AUC), a statistic that has no tunable parameters and measures the ability of a classifier to correctly rank independent positive and negative examples, we saw no difference between CNNs trained on broad-band and band-limited data (**Fig H**).

As we have discussed in the main text, this apparent discrepancy between classifier accuracy and the AUC when applied to band-limited data is simply because the measure different things, and suggests that ML-based methods should be used with care when used to detect the presence (and absence) of information in electrical recordings.

Fig G. **Classifier accuracy on band-limited data.** **(A)** Left: Mean Spike-Triggered Average (mSTA) traces for “airflow” (blue) and “touch” (purple) computed for ±80 ms relative to spikes occurring during baseline. mSTA is computed by averaging STAs over all cells in the same nucleus. The number of visible lines corresponds to the simultaneously recorded stable cells in each nucleus used to compute mSTA. Pale lines are single STA traces and dark lines are the mean STA. Right: Comparison of average power spectra of STA traces for airflow and grooming blocks. The two spectra show differences in the 10-17 Hz (yellow) and 17-25 Hz (blue) bars. **(B)** A trial spectrogram illustrating the power in the 10-17Hz (yellow) and 17-25Hz (blue) frequency bands. **(C)** Accuracy of CNNs trained on spectrograms restricted to only 10-17 Hz (yellow), 17-25Hz (blue) and 10-25 Hz (green) bands. Plots are organized by nucleus (rows: Central, Basal, Accessory Basal, Lateral) and recording session (columns), with number of recording contacts in each nucleus displayed in the top left corner. The horizontal black dotted line represents the mean accuracy of the network trained on the full spectrogram. The horizontal blue line represents theoretical chance at 50%. The gray bars correspond to 1 and 2 standard deviations about the mean.

​​

Fig H. **AUC show no distinct difference between network discriminability using broadband versus band-limited data.** AUROC of CNNs trained on spectrograms restricted to only 10-17 Hz (yellow), 17-25Hz (blue) and 10-25 Hz (green) bands. Plots are organized by nucleus (rows: Central, Basal, Accessory Basal, Lateral) and recording session (columns), with number of recording contacts in each nucleus displayed in the top left corner. The horizontal black dotted line represents the mean accuracy of the network trained on the full spectrogram. The horizontal blue line represents theoretical chance at 50%. The gray bars correspond to 1 and 2 standard deviations about the mean.

**Autonomic state difference in airflow versus grooming blocks.** (A) Mean heart rate measurements during airflow and grooming blocks for Monkey A (left), Monkey S (center), and Monkey C. (B) Mean RSA strength during airflow and grooming blocks for Monkey A (left), Monkey S (center), and Monkey C.
